# Application of Cryopreserved Human Hepatocytes in Trichloroethylene Risk Assessment: Relative Disposition of Chloral Hydrate to Trichloroacetate and Trichloroethanol

**DOI:** 10.1289/ehp.9047

**Published:** 2006-05-30

**Authors:** Apryl Bronley-DeLancey, David C. McMillan, JoEllyn M. McMillan, David J. Jollow, Lawrence C. Mohr, David G. Hoel

**Affiliations:** 1 Department of Biostatistics, Bioinformatics and Epidemiology; 2 Department of Cell and Molecular Pharmacology and; 3 Department of Medicine, Medical University of South Carolina, Charleston, South Carolina, USA

**Keywords:** alcohol dehydrogenase, aldehyde dehydrogenase, chloral hydrate, genetic variability, human hepatocytes, metabolism, risk assessment, trichloroacetate, trichloroethylene

## Abstract

**Background:**

Trichloroethylene (TCE) is a suspected human carcinogen and a common ground-water contaminant. Chloral hydrate (CH) is the major metabolite of TCE formed in the liver by cytochrome P450 2E1. CH is metabolized to the hepatocarcinogen trichloroacetate (TCA) by aldehyde dehydrogenase (ALDH) and to the noncarcinogenic metabolite trichloroethanol (TCOH) by alcohol dehydrogenase (ADH). ALDH and ADH are polymorphic in humans, and these polymorphisms are known to affect the elimination of ethanol. It is therefore possible that polymorphisms in CH metabolism will yield subpopulations with greater than expected TCA formation with associated enhanced risk of liver tumors after TCE exposure.

**Methods:**

The present studies were undertaken to determine the feasibility of using commercially available, cryogenically preserved human hepatocytes to determine simultaneously the kinetics of CH metabolism and ALDH/ADH genotype. Thirteen human hepatocyte samples were examined. Linear reciprocal plots were obtained for 11 ADH and 12 ALDH determinations.

**Results:**

There was large interindividual variation in the *V*_max_ values for both TCOH and TCA formation. Within this limited sample size, no correlation with ADH/ALDH genotype was apparent. Despite the large variation in *V*_max_ values among individuals, disposition of CH into the two competing pathways was relatively constant.

**Conclusions:**

These data support the use of cryopreserved human hepatocytes as an experimental system to generate metabolic and genomic information for incorporation into TCE cancer risk assessment models. The data are discussed with regard to cellular factors, other than genotype, that may contribute to the observed variability in metabolism of CH in human liver.

Trichloroethylene (TCE), a common metal degreasing solvent, is considered to be the major component in more than half the U.S. Environmental Protection Agency hazardous waste sites (Fay and Mumtaz 1996). Although rodent bioassay studies have established that exposure to TCE is associated with development of neoplasia in a variety of organ systems (Davidson and Beliles 1991), epidemiologic studies have not provided support for analogous susceptibility of humans (Lewandowski and Rhomber 2005; Wartenburg et al. 2000). Efforts to redefine the risk of TCE-induced carcinogenicity have been driven by the ability of this solvent to induce hepatocellular carcinoma in the B6C3F_1_ mouse strain (Herren-Freund et al. 1987). It is now well accepted that hepatocarcinogenicity is caused by a metabolite, trichloroacetate (TCA), although a contribution by its dichloroacetate (DCA) analog cannot be discounted at this time (Bull 2000; Bull et al. 2002).

TCE is metabolized in the liver (Figure 1), predominately by cytochrome P450 enzyme CYP2E1 (Lipscomb et al. 1997; Nakajima et al. 1992), to chloral hydrate (CH). In turn, CH is metabolized by either of two pathways: oxidation to TCA catalyzed by aldehyde dehydrogenase (ALDH), or reduction to a noncarcinogenic metabolite, trichloroethanol (TCOH), mediated by alcohol dehydrogenase (ADH). Although the conversion of CH to TCOH is considered to be reversible, the extent to which this back-reaction contributes to TCA pools *in vivo* is unclear. In the main, the distribution of CH into its carcinogenic (TCA) and noncarcinogenic (TCOH) metabolites appears to depend on the relative activity of the two pathways.

Animals and humans appear to handle TCE in regard to absorption, distribution, metabolism, and elimination in a qualitatively similar fashion. It is thus attractive to use physiologically based pharmacokinetic (PBPK) models for dose and species extrapolation. Several models have been developed for this purpose using kinetic parameters (e.g., *K_m_* and *V*_max_) derived from both human and animal studies (Clewell and Andersen 2004; Fisher 2000).

It is well known that both ADH and ALDH are polymorphic in humans and that the isoforms may differ markedly in their steady-state kinetic constants (Li et al. 2001). The human hepatic ADH concerned with the metabolism of ethanol is designated as class I and is encoded by three genes, *ADH1*, *ADH2*, and *ADH3*, which code for peptides α, β, and γ (Ehrig et al. 1990; Xu et al. 1988; Yin et al. 1984). Because the active enzyme is a dimer, human hepatic ADH may occur as any of 21 possible forms depending on the individual’s genotype. Among Caucasians, β_1_ predominates (80–95%) over β_2_ (4–20%). Asians show a reverse pattern (β_1_ ~ 30% and β_2_ ~ 70%). About 25% of African Americans express the β_3_ form of ADH in their livers. Of importance for the present study, the kinetic parameters *V*_max_ and *K_m_* toward ethanol vary considerably among the isomeric forms, with a resultant range of about a 50-fold difference in their first-order rate constants (*V*_max_/*K_m_*). At ethanol blood levels associated with intoxication (10–20 mM), the rate of elimination of ethanol in the liver appears to be most closely associated with the *V*_max_ of the individual’s ALDH isoform and with the rate of regeneration of the essential cofactor NAD^+^ from its reaction product, NADH. Regarding CH, the situation is less clear. At the low levels of TCE in drinking water, the steady-state production of CH may be very low, with corresponding low levels of CH in the liver. Theoretically, the first-order rate constant for CH metabolism would be the prime determinant of elimination by this pathway.

ALDH is also polymorphic (Ehrig et al. 1990). For acetaldehyde metabolism, *ALDH1* (cytosolic) and *ALDH2* (mitochondrial) appear to be the most important, with the mitochondrial form contributing most of the clearance. An aberrant (inactive) form of *ALDH2* occurs in many individuals of Asian descent and has been considered to be responsible for their lesser ability to metabolize acetaldehyde generated from ethanol by ADH. ALDH is active as a tetramer, and the presence of even one inactive monomer in the tetramer significantly impairs acetaldehyde elimination and results in a “flushing” reaction and nausea in these individuals after ethanol ingestion.

Collectively, consideration of the known polymorphisms of ADH and ALDH in humans raises the possibility of significant variation in the contribution of the two pathways (conversion to TCOH by ADH or to TCA by ALDH) in the elimination of CH formed in the liver from TCE ingested in drinking water. Because hepatocarcinogenicity is considered to be related to TCA levels in the liver, this variation could contribute significantly to relative susceptibility among exposed humans.

The question of relative distribution of CH to TCOH and TCA was examined by Lipscomb et al. (1996) using 700 × *g* supernatant fractions of homogenized livers from humans, mice, and rats. They reported that in all three species, TCOH was the major metabolite when concentrations of CH were below 1 mM. However, these studies were performed by incubating CH/liver homogenate separately with either NAD^+^ (for TCA formation) or NADH (for TCOH formation) at optimal concentrations (0.9 mM) of nucleotide. As discussed below, these experimental conditions may not reflect the environment of the intact hepatocyte, so it is unclear whether kinetic constants obtained *in vitro* are predictive of the *in vivo* situation.

Cryopreserved human hepatocytes are now readily available from commercial sources and hence offer the possibility of rapid assessment of a large number of individuals with varying genotypes. The present studies were undertaken to determine whether cryopreserved hepatocytes could be used to examine the distribution of CH into its carcinogenic and noncarcinogenic metabolites and, if so, whether the activities of each pathway could be correlated with ADH and ALDH genotypes. The data indicate that cryopreserved hepatocytes readily metabolized CH and that the data were amenable to Lineweaver-Burke kinetic analysis. Although the individual samples showed major differences in activity for both pathways, the ratio of oxidation to reduction was relatively constant. In view of the relatively small number of human samples (i.e., 13) in these initial studies, no correlation could be made between enzymatic activities and ADH/ALDH genotype. We also discuss the possibility that factor(s) other than ADH/ALDH genotype may influence activity at low substrate concentrations.

## Materials and Methods

### Chemicals and materials

Cryopreserved human hepatocytes were purchased from InVitro Technologies (Baltimore, MD) and ZenBio (Research Triangle, NC). The cells were stored in liquid N_2_ until use. InVitroGRO HI incubation medium and Torpedo antibiotic mix were purchased from InVitro Technologies. CH, TCA, DCA, TCOH, RNase A, and ethidium bromide were obtained from Sigma Chemical Co. (St. Louis, MO). The DNeasy tissue kit was obtained from Qiagen Inc. (Valencia, CA). Eppendorf hot master mix was obtained from Fisher Scientific (Freehold, NJ). Bovine serum albumin (BSA) was purchased from Promega Scientific (Madison, WI). ALDH and ADH primers and restriction enzymes were obtained from Integrated DNA Technologies (Coralville, IA). Ten percent TBE (Tris-borate-EDTA) Novex gels and TBE-running buffer were purchased from Invitrogen (Carlsbad, CA). All other reagents were analytical grade and purchased from commercial sources.

### Metabolism studies

Just before use the hepatocytes were thawed according to the suppliers’ instructions and counted; cell viability was determined by trypan-blue exclusion. Hepatocytes were diluted to a concentration of 1 × 10^6^ cells/mL with InVitro Technologies HI incubation medium containing Torpedo antibiotic mix and transferred to reaction vials in 0.5-mL aliquots. CH (0.06–2.5 mM) was added in a small volume of incubation medium to each vial. The cells were incubated with CH for 10 min at 37°C with gentle shaking. After the incubation period, three 10-μL aliquots were withdrawn from each vial and placed in a 20-mL gas chromatography (GC) vial containing 200 μL esterizer (H_2_O/H_2_SO_4_/methanol, 6:5:1) to enable volatilization of the acetates. The esterized samples were analyzed by gas chromatography (GC) with electron-capture detection for the presence of CH metabolites.

The reproducibility of this experimental method was assessed in liver homogenates (700 × *g* supernatant) prepared from an untreated male Sprague-Dawley rat (five separate suspensions of the same liver) and untreated male B6C3F_1_ mice (four suspensions from four individual male mice of the same age). Excision of livers from anesthetized animals was carried out in accordance with the *Guide for the Care and Use of Laboratory Animals* (Institute of Laboratory Animal Resources 1996) guidelines as adopted by the National Institutes of Health. Frozen (–80°C) pieces of these livers were thawed and homogenized in 4 vol of homogenization buffer (250 mM sucrose, 25 mM KCl, 1 mM dithiothreitol, 0.5 mM EDTA, 10 mM HEPES, 20% glycerol) and centrifuged at 700 × *g* for 10 min at 4°C. The supernatant was removed and kept on ice until use. Protein content of the supernatant was determined as described previously (Smith et al. 1985). NAD^+^, NADP^+^ (0.9 mM), and various concentrations of CH were added to 0.5 mL of the supernatant. The reactions were carried out at 37°C for 10 min. Aliquots of each reaction mixture (10 μL) were prepared for GC analysis as described above for human hepatocytes. In each case, the experimental variability did not exceed 10%.

GC analysis of CH metabolites was performed essentially as described previously (Muralidhara and Bruckner 1999). Samples were analyzed on a PerkinElmer Autosystem XL gas chromatograph fitted with a headspace autosampler and an electron-capture detector (PerkinElmer, Inc., Wellesley, MA). Metabolites were resolved using a 10 inch × 1/8 inch outer-diameter stainless-steel column packed with 10% OV-17 on Supelcoport (Supelco, Bellefonte, PA). The temperatures for analyses were as follows: the column was run isothermal at 150°C, injector at 200°C, transfer line at 120°C, and detector at 360°C. Nitrogen was used as the carrier gas at 60 mL/min with a headspace pressure of 20 psi. The samples were heated at 110°C in the autosampler chamber for 30 min before injection into the GC; run time was 8 min. The metabolites were quantified against a standard curve using authentic DCA, TCOH, and TCA (10–1,000 ng).

### ADH and ALDH genotyping

Determination of ADH and ALDH genotype was performed on the same cryopreserved hepatocytes that were used for the metabolism studies. Detection of *ADH2*, *ADH3*, and *ALDH2* was performed using previously described methods [Nakamura et al. 1993; human alcohol dehydrogenase (*ADH*; alcohol:NAD^+^ oxidoreductase, EC 1.1.1.1), UniGene accession no. P07327 (http://www.ncbi.nlm.nih.gov/entrez/query.fcgi?CMD=search&DB=unigene); human (mitochondrial) aldehyde dehydrogenase (*ALDH2*), EC 1.2.1.3, UniGene accession no. P05091; and human (cytosolic) aldehyde dehydrogenase (*ALDH1*), EC 1.2.1.3, UniGene accession no. P00352]. DNA was isolated from approximately 5 × 10^5^ cells using a Qiagen DNeasy Tissue Kit with the RNase A step. Polymerase chain reaction (PCR) amplification of the *ADH* and *ALDH* alleles was performed using the primers and restriction enzymes listed in Table 1. Primers were constructed based upon previously reported sequences (Burnell et al. 1989; Ehrig et al. 1990; Mulligan et al. 2003; Osier et al. 1999; Yin et al. 1984). Each reaction mixture contained 20 μL Eppendorf hot master mix, 1 μL of 5 μM forward primer, 1 μL of 5 μM reverse primer, 17 μL sterile water, and 10 μL isolated DNA template. PCR was carried out using the following conditions: 4 min at 95°C, 1 min at 95°C, 1 min at 55°C, 1 min at 72°C, second and fourth steps cycled 34 times, 30 min at 72°C, and hold at 4°C.

The PCR products were isolated from the whole DNA by restriction enzyme digest. Briefly, to the 20 μL PCR reaction mixture we added 5.5 μL H_2_O (RNase, DNase free), 3 μL buffer, 1 μL restriction enzyme, and 0.5 μL BSA. The reaction was carried out at 37°C for 2–3 hr. The digest product (10 μL) was separated by gel electrophoresis using a 10% TBE Novex gel and visualized with ethidium bromide. The gel was run for 20 min at 200 V to obtain optimal PCR fragment separation. A 100-bp DNA ladder was run concurrently with the samples for determination of product molecular weight. The gels were photographed for analysis using Biorad Quantity One ChemDoc software (BioRad, Hercules, CA).

### Statistical analysis

Data were analyzed using Stata software (version 8.2; StataCorp LP, College Station, TX), and robust regressions were performed to remove outliers. The “rreg” command was used to fit the regression model; the final kinetic values were determined using this method. SAS software (version 9.0; SAS Institute Inc., Cary, NC) was used to determine the reproducibility of the assay procedure that employed the mouse and rat liver homogenates.

## Results

### CH metabolism by liver homogenates: species comparison

Appreciable species-related differences have been reported for the kinetic constants of CH metabolism into TCOH and TCA by liver homogenates (Lipscomb et al. 1996). To validate our experimental assay procedure, we examined the capacity of rat and mouse liver homogenates to metabolize CH to TCOH and TCA. The kinetic constants (*K_m_* and *V*_max_) obtained for the conversion of CH to TCOH (Table 2) were in the 100- to 400-μM range and were similar to values reported previously.

The *K_m_* values obtained for TCA formation were in the 100- to 200-μM range and comparable to those seen for TCOH formation by the same liver fraction. Although these values are notably lower than those observed by Lipscomb et al. (1996), it is of interest that the calculated first-order rate constants (*V*_max_/*K_m_*) for TCA formation showed the same species rank order: mouse > human > rat.

### CH metabolism in isolated human hepatocytes

Because metabolism of CH to TCA and TCOH by liver homogenates requires addition of NAD^+^ and NADH, respectively, the activity of each pathway must be examined in separate incubations. To avoid the complication introduced by the different reaction conditions in the assessment of the distribution of CH formed in the liver after ingestion of TCE, we examined the suitability of isolated human hepatocyte preparations for this purpose. Cryogenically preserved human hepatocytes (~ 10 × 10^6^ cells/sample) were obtained commercially, thawed, and equilibrated in the recovery buffer as recommended by the supplier. Cell viability was then determined, and the cells were resuspended to give a final density of 1 × 10^6^ viable cells/mL of incubation mixture.

Preliminary studies established that the metabolism of CH in the hepatocyte suspensions was linear with time for 10 min at 37°C over a concentration range of 0.05–2.0 mM. The double-reciprocal plot of the simultaneous metabolism of CH to TCOH and TCA by one human hepatocyte sample (CHD) is illustrated in Figure 2.

Table 3 lists the demographic and lifestyle characteristics of the donors of the 13 human hepatocyte samples supplied by the vendors. Nonlinear kinetics was observed in the formation of TCOH in two individuals (HL7 and HL8), and for TCA formation in one individual (HL6). The reason for nonlinear kinetics in these samples is unknown, although this phenomenon has been described previously using ethanol as a substrate (Dalziel and Dickinson 1966). The apparent kinetic constants for the other individual hepatocyte samples and their genotypic forms of ADH and ALDH are shown in Table 4. The means of these values (*K_m_* of 0.06 ± 0.18 and 0.12 ± 0.03 mM, and *V*_max_ of 75.3 ± 38.4 and 53.9 ± 26.2 nmol/min/mg, respectively, for TCOH and TCA formation) are generally similar to those previously reported for the 700 × *g* fraction of human liver homogenates (Lipscomb et al. 1996). Of note, however, is the wide variation in the derived *K_m_* and *V*_max_ values among individuals for both TCOH and TCA formation.

Because the two pathways may be regarded as competing for CH, it was of interest to compare the relative activity of the two pathways in the various individuals. Figure 3 shows a plot of the *V*_max_ for TCOH formation against the *V*_max_ for TCA formation for each of the hepatocyte preparations. The values appear to cluster along the diagonal of the graph, indicating that increase in one activity is matched by increase in the other. This relationship suggests that, although the activity of each pathway varies significantly among individuals, the disposition of CH into the two metabolites is constant. This relationship was further examined by calculation of the first-order rate constants (*V*_max_/*K_m_*) for the two enzymatic pathways. Figure 4 shows the plot of the first-order rate constants for TCOH versus TCA. Although less striking than those for the *V*_max_ plots, there appears to be a similar tendency to cluster along the diagonal, again indicating that the disposition of CH into the two pathways among the individual samples is relatively constant regardless of the large variation in activity of the two individual pathways.

## Discussion

It is now well appreciated that humans vary considerably in susceptibility to environmental toxicants and that this variability is of major concern in assessing the risk posed by hazardous chemicals for human health (Aldridge et al. 2003). The factors determining variability include genetics, age, sex and disease state, both alone and in combination. The complexity of the situation is further compounded by the need to extrapolate from high-dose levels used in rodent bioassays to chronic low-dose levels typical of drinking water exposure in humans. Currently, uncertainty factors may be used to account for human variability, and “average” kinetic values are used in PBPK models for dose extrapolation. Clearly, our ability to account for human variability in response to hazardous chemicals would be greatly improved if arbitrary uncertainty factors could be replaced by quantitative measurement and the “average” kinetic constants by values appropriate to the various populations at risk (Gentry et al. 2002).

Obtaining kinetic data from prospective clinical studies to encompass the range of human genetic variability clearly presents major difficulties. The growing availability of cryogenically preserved human hepatocytes could partially solve this dilemma in that it may be practical to perform kinetic and genetic analyses on a large number of donors in a rapid and cost-effective manner. The present studies were undertaken to determine the feasibility of using cryopreserved cells as a sampling source to probe the relationship between genotype and metabolic disposition into toxic versus nontoxic pathways of metabolism. CH was chosen for this purpose because *a*) it is a known intermediate in the pathway that generates a carcinogenic metabolite (TCA) from an environmental contaminant (TCE); *b*) it is cleared competitively by two pathways, one leading to TCA and the other to the non-carcinogenic metabolite TCOH; and *c*) the enzymes concerned with its clearance (ALDH/ ADH) are polymorphic in humans. Because the objective was to determine if the cells could be used immediately after thawing, no recovery or passage procedures were used beyond the suppliers’ instructions.

Experimentally, 13 samples of cryopreserved human hepatocytes were obtained commercially, thawed and reconstituted as directed, and incubated with various concentrations of CH. Metabolite formation was quantitated and used to generate the kinetic constants *K_m_* and *V*_max_ (Figure 2). These cells were also used to determine ADH and ALDH genotypes.

As shown in Table 4, nine of the samples had the *ADH2*β_1_β four were *ADH2* β_1_β_2_. Nine samples were *ADH3*γ_1_γ_1_ and four were *ADH3*γ_1_γ_2_. In regard to metabolism, two samples (HL7 and HL8) yielded nonlinear kinetics for TCOH formation and were excluded from calculation. The basis for the aberrant kinetic behavior is unknown. The remaining values gave mean *K_m_* and *V*_max_ values of 0.06 ± 0.018 mM and 75.3 ± 38.4 nmol/min/10^6^ cells, respectively. In view of the limited representation of β_2_ and γ_2_ forms of the isoforms within the samples, no conclusions could be drawn as to the influence of genotype on susceptibility to TCE hepatocarcinogenicity.

A similar situation was seen with the ALDH kinetic parameters. All but two of the samples had the same genotype (*ALDH2* *1). The two samples that were not *ALDH2* *1 were termed “nonreacting”; that is, the phenotype was not that of the inactive form associated with acetaldehyde intolerance. An additional sample showed nonlinear kinetics. Within the series, the mean values for *K_m_* and *V*_max_ were 0.12 ± 0.03 mM and 53.9 ± 26.2 nmol/min/10^6^ cells, respectively.

The most striking aspect of these results is the large variation among the individual samples regardless of genotype. For both ADH and ALDH, the *V*_max_ values showed more than a 10-fold variation, raising the possibility that the uncertainty factor of 10 commonly used to assess human variability may be an underestimation.

**Figure 1:**
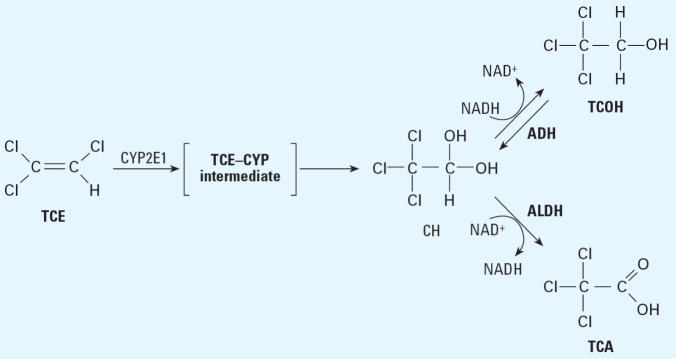
Metabolism of TCE to CH and subsequent disposition into its carcinogenic (TCA) and noncarcinogenic (TCOH) pathways.

**Figure 2:**
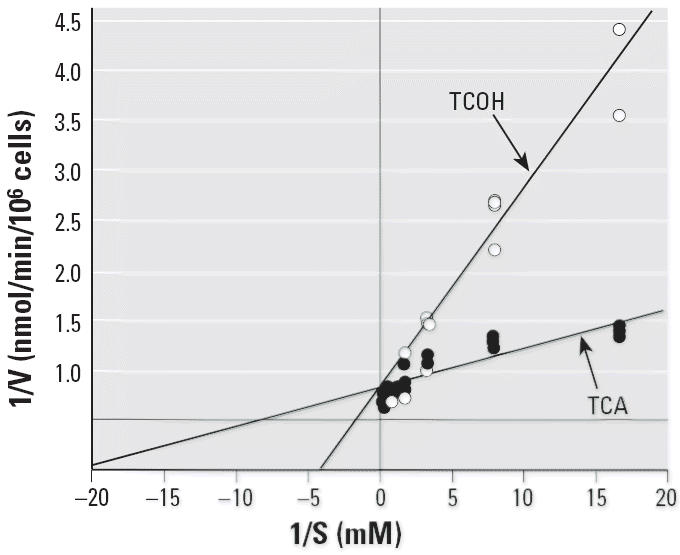
Lineweaver-Burke plot of the raw data points (*n* = 3) for TCOH and TCA formation in a suspension of one human hepatocyte sample (CHD).

**Figure 3:**
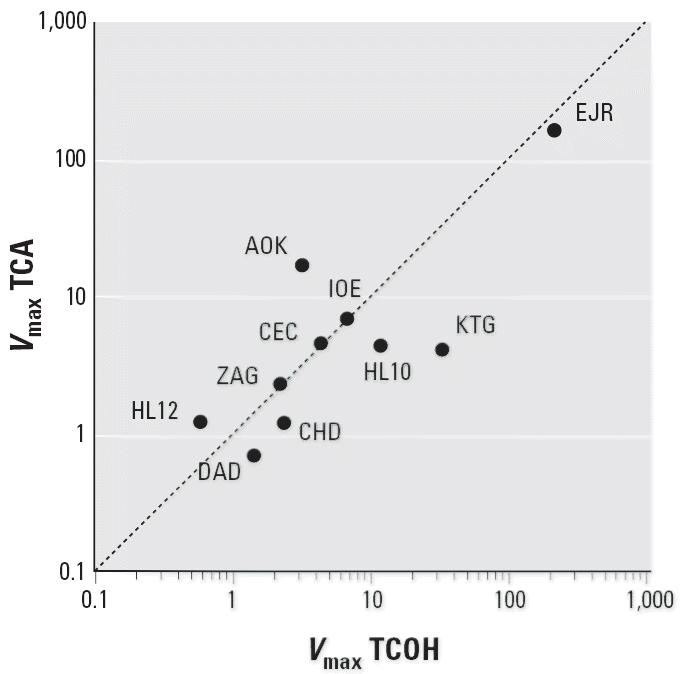
Plot of *V*_max_ versus *V*_max_ for ADH and ALDH activities in human hepatocyte suspensions given CH. *V*_max_ values were determined from linear regression analysis of the raw data points.

**Figure 4:**
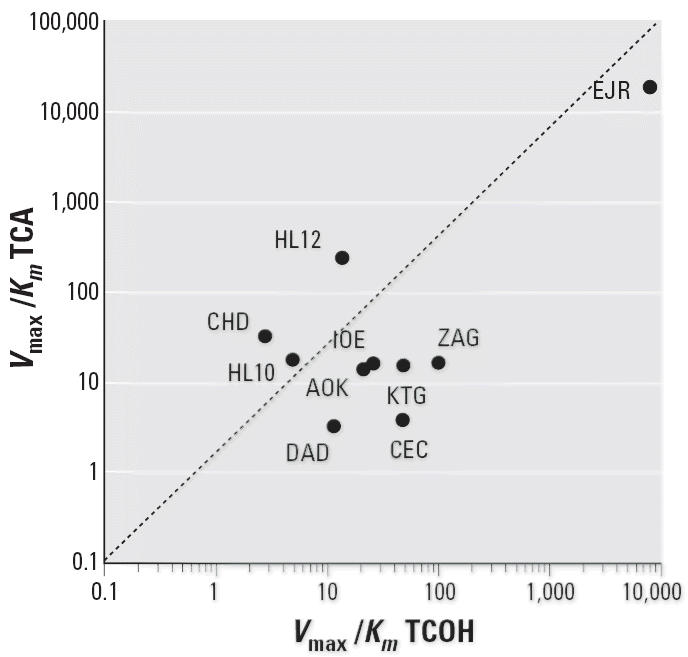
Plot of first-order rate constants (*V*_max_/*K_m_*) for ADH versus ALDH activities in human hepatocyte suspensions given CH.

However, as illustrated in Figure 1, production of TCA from CH depends on the ratio of the activities of both ADH and ALDH rather than on ALDH alone. Accordingly, the *V*_max_ values for each pathway were plotted against each other for each individual sample (Figure 3). Although there was a significant difference in the activities of the pathways among the samples, it is noteworthy that their ratios fall reasonably close to the diagonal. Of interest, the sample EJR, which was an outlier for both pathways, also showed a ratio that approximated unity. Although not as striking, a similar relationship could be observed when the first-order rate constants (*V*_max_/*K_m_*) for the two pathways were plotted against each other (Figure 4). Collectively, these data suggest that in spite of large differences in the activities of the individual pathways, the distribution of CH into its noncarcinogenic and carcinogenic metabolites may be relatively constant.

The reason for the large variability in ADH and ALDH activity among these samples is not known. However, a plausible explanation may lie in the steady-state kinetics of CH elimination. It has long been known that ADH-catalyzed oxidation of ethanol/reduction of acetaldehyde is an equilibrium reaction in which the direction and rate depend on both the concentration of substrate (alcohol or aldehyde) and the concentration and form of the pyridine cofactor (i.e., NAD^+^ or NADH). The reaction has been described in terms of eight kinetic constants (Dalziel and Dickinson 1966). Although the reaction yields linear double-reciprocal plots and is hence amenable to Lineweaver-Burke analysis, the kinetic parameters derived are simplifications of the overall Theorell-Chance mechanism (Theorell and Chance 1951). Special cases exist where the reciprocal plots are not linear. Of importance for the present studies, the reaction appears to be ordered with addition of pyridine nucleotide preceding ethanol and the release of NADH being the rate-limiting step (Ehrig et al. 1990). Thus, the availability and form of the cofactor can explain differences in the *V*_max_ of the reaction. Differences in *K_m_* values are less well understood but may reflect inclusion of a catalytic constant (*K*_cat_) of ethanol oxidation/ acetaldehyde reduction in experimentally observed values.

**Figure 5:**
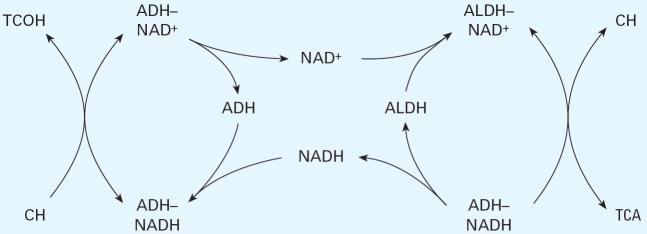
Proposed interdependence of oxidation and reduction of CH by ADH and ALDH (see “Discussion” for description).

For the present studies, a simplified schema appears adequate to explain most of the observed activities and the apparent constant ratio between the two pathways. As shown in Figure 5, reduction of CH by the NADH/ ADH complex yields TCOH with subsequent release of NAD^+^. The NAD^+^ would then be available to react with ALDH, and the complex would oxidize CH to TCA with subsequent release of NADH. Thus, the reduction and oxidation of CH by ADH and ALDH would be independent of the initial NAD^+^:NADH ratio. This interdependence may normalize the ratio of oxidation and reduction, thus yielding the observed approximate linear correlation between the two pathways. This proposed mechanism implies that there is no kinetic barrier for pyridine nucleotide exchange between the two enzymatic pathways, suggesting that both enzymes are located in the same cellular compartment. This situation would be satisfied if the ALDH used for CH oxidation was the cytosolic isoform (*ALDH1*) rather than the mitochondrial isoform (*ALDH2*), which is believed to be more important for the elimination of acetaldehyde generated from ethanol. Future studies are needed to probe the role of cellular pyridine nucleotide levels in the apparent *K_m_* and *V*_max_ values and to distinguish the contribution of the two ALDH isoforms to CH oxidation by their sensitivity to disulfiram (Ehrig et al. 1990).

Previous studies on the relative disposition of CH into its oxidative and reductive pathways used liver homogenates (Lipscomb et al. 1996). Each pathway was measured in separate incubations with optimal pyridine nucleotide concentrations and a concentration range of CH of up to 20 mM. These investigators concluded that TCOH formation predominated at submillimolar concentrations of CH. The unequal disposition of CH with preponderance of TCOH formation has also been observed in experimental animals. Abbas et al. (1996) administered CH to B6C3F_1_ mice at doses of 10–300 mg/kg and reported an approximate 3:1 ratio for the first-order rate constants for TCOH versus TCA formation. The present studies relied on endogenous levels of NAD^+^ and NADH within the hepatocytes and used a CH substrate concentration range of 0.060–2.0 mM. Additional studies are warranted to determine whether the approximate 1:1 ratio seen in these studies reflects the low level of CH substrate or a low level of pyridine nucleotide in the cryopreserved hepatocytes. It is also possible that hepatocellular pyridine nucleotide levels in humans are under genetic control and that at very low levels of CH generated from drinking water levels of TCE, the genetically determined concentration of pyridine nucleotides may be more important than the ADH/ALDH genotypes of the exposed individual.

Collectively, the present studies show that cryogenically preserved hepatocytes can provide a cost-effective approach to the simultaneous determination of the kinetics of elimination of CH by ADH and ALDH and the determination of ADH and ALDH genotypes of individual donors. Both metabolism and genotyping can be done on as few as 10^7^ cells. Application of this approach to a larger number of ethnically diverse donor samples would allow for definition of the relationship between genotype and susceptibility to TCE-induced liver tumors. However, the studies also raise a number of fundamental questions, such as which genes are important at very low (i.e., drinking water) levels of exposure to environmental chemicals. Is it the isoform of the metabolizing enzyme or of a “housekeeping” gene that sets the physiologic homeostasis of the cell? Genes that control nucleotide formation and removal would fall into this category. It is also important, however, to characterize the cryogenically preserved cells in greater detail. Although the recovered cells in this study all showed high viability, and their metabolic activity is expressed in terms of viable cell number, it is possible that their physiologic state may vary in ways that affect the kinetic interpretation of the data for PBPK modeling and subsequent risk assessment. Additional studies directed at normalizing the cellular physiologic homeostasis of individual donor samples before experimentation would allow distinction between genetically endowed differences and treatment/ handling effects.

The 11 individuals in the present study who showed the 2*1 polymorphism for ALDH2 had a *V*_max_ variability for the pathway generating TCA of between one and two orders of magnitude (Table 4). When the two non-2*1 genotypic individuals were included, the range of *V*_max_ values was increased by an additional order of magnitude. This observation may have implications for quantitative risk estimation for environmental exposures to TCE in the general population. In risk assessment a default value of 10 is used as an uncertainty factor for genetic variability in the population. The results of this study indicate that if *V*_max_ is used as the appropriate correlate to risk, then a 10-fold uncertainty factor is an underestimate. For TCE, however, what is relevant in risk assessment is the amount of TCA, which depends on the *V*_max_ values for TCA metabolism as well as the *V*_max_ for the competitive pathway to TCOH. Thus, Figure 3 is the relevant comparison and the *V*_max_ values for TCA and TCOH are about equal, at least within an order of magnitude for all the samples. At lower exposures, a comparison of the rate constants (*V*_max_/*K_m_*) for the two competing pathways would be relevant. Figure 4 shows a good correlation with the two rate constants being approximately within an order of magnitude of each other. This suggests that a 10-fold uncertainty factor for genetic susceptibility is reasonable for TCA-driven TCE carcinogenesis. Further research is warranted to quantify the genetic variation in liver cancer risk following environmental TCE exposures due to the polymorphic genes involved in its metabolism.

## Figures and Tables

**Table 1:** Primer sequences and restriction enzymes used in ALDH and ADH genotyping.

Isoform (primer ID)	Primer sequence	Restriction enzyme
ALDH2 (YC3)	5′-TTG GTG GCT AGA AGA TGT C-3′	*MboII*
ALDH2 (YC4)	5′-CCA CAC TCA CAG TTT TCT CTT-3′	*MboII*
ADH2 (A2F)	5′-ATT CTA AAT TGT TTA ATT CAA GAA G-3′	*MsII*
ADH2 (A2R)	5′-ACT AAC ACA GAA TTA CTG GAC-3′	*MsII*
ADH2 (424)	5′-TGG ACT CTC ACA ACA AGC ATG GT-3′	*AluI*
ADH2 (290)	5′-TTT CTT TGG AAA GCC CCC AT-3′	*AluI*
ADH2 (352)	5′-TCT TTC CTA TTG CAG TAG C-3′	*AluI*
ADH3 (321)	5′-GCT TTA AGA GTA AAT ATT CTG TCC CC-3′	*SspI*
ADH3 (351)	5′-AAT CTA CCT CTT TCC GAA GC-3′	*SspI*

**Table 2:** Kinetics of TCOH and TCA formation from CH in 700 × *g* supernatants obtained from present data compared with data in the literature.

	Present data		Lipscomb et al. (1996) data	
	*V*_max_	*K_m_*	*V*_max_	*K_m_*
TCOH				
Rat	9.1	0.37	24.3	0.52
Mouse				
High affinity	8.9	0.11	6.3^a^	0.12
Low affinity			6.1	0.51
Human	ND	ND	34.7	1.34
TCA				
Rat	2.5	0.20	4.0	16.41
Mouse	8.9	0.10	10.6	3.50
Human	3.9	0.17	65.2	23.90

ND, not determined. *V*_max_ is expressed as nmol/min/mg 700 × *g* supernatant protein. *K_m_* is expressed as mM CH.

a Inhibited above 0.57 mM CH.

**Table 3:** Donor characteristics of cryopreserved human hepatocytes.

Cells^a^	Donor age (years)	Sex	Race	Tobacco use	Alcohol use	Substance abuse
AOK	47	M	C	Y	N	N
CEC	48	M	C	Y	Y	Y
DAD	62	M	C	Y	Y	N
IOE	79	M	C	Y	Y	N
KTG	59	M	C	Y	Y	N
ZAG	59	M	C	Y	Y	N
CHD	72	F	AA	N	N	N
EJR	56	F	C	N	N	N
HL10	42	M	AA	Y	Y	Y
HL6	48	F	C	N	N	N
HL7	55	F	C	N	N	N
HL8	56	M	C	Y	Y	N
HL12	0.03	F	C	N	N	N

Abbreviations: AA, African American; C, Caucasian; F, female; M, male; N, no; Y, yes.

a Viability of hepatocytes when thawed was 84.6 ± 2.6% (mean ± SE).

**Table 4:** Kinetics of TCOH and TCA formation in relation to ADH and ALDH genotypes.

	TCOH				TCA		
Donor	*K_m_*	*V*_max_	*ALDH_2_*	*ALDH_3_*	*K_m_*	*V*_max_	*ALDH_2_*
HL12	0.043	0.590	β_1_β_1_	γ_2_γ_2_	0.005	1.200	2*1
CHD	0.829	2.360	β_1_β_1_	γ_1_γ_2_	0.040	1.220	2*1
KTG	0.700	33.330	β_1_β_1_	γ_1_γ_1_	1.130	4.150	2*1
HL10	2.440	12.070	β_1_β_1_	γ_1_γ_1_	0.240	4.350	2*1
CEC	0.093	4.420	β_1_β_1_	γ_1_γ_1_	0.294	4.590	2*1
IOE	0.270	6.990	β_1_β_1_	γ_1_γ_1_	0.410	6.760	2*1
HL6	0.001	0.540	β_1_β_1_	γ_1_γ_2_	ND	ND	2*1
HL7	ND	ND	β_1_β_1_	γ_1_γ_1_	0.013	0.140	2*1
AOK	0.160	3.340	β_1_β_1_	γ_1_γ_1_	1.270	16.940	Nonreacting
HL8	ND	ND	β_1_β_2_	γ_1_γ_1_	0.329	0.410	2*1
DAD	0.130	1.450	β_1_β_2_	γ_1_γ_1_	0.220	0.710	2*1
ZAG	0.022	2.220	β_1_β_2_	γ_1_γ_1_	0.142	2.280	2*1
EJR	0.027	222.220	β_1_β_2_	γ_1_γ_1_	0.009	158.730	Nonreacting
Mean ± SE	0.060 ± 0.018	75.30 ± 38.36			0.124 ± 0.031	53.91 ± 26.21	

ND, not determined. *K_m_* is expressed as mM CH; *V*_max_ is expressed as nmol/min/mg cell protein.
